# Endotoxin emissions from commercial composting activities

**DOI:** 10.1186/1476-069X-8-S1-S9

**Published:** 2009-12-21

**Authors:** Lewis Deacon, Louise Pankhurst, Jian Liu, Gillian H Drew, Enda T Hayes, Simon Jackson, James Longhurst, Philip Longhurst, Simon Pollard, Sean Tyrrel

**Affiliations:** 1 Centre for Resource Management and Efficiency, Sustainable Systems, School of Applied Sciences, Cranfield University, Cranfield, Bedfordshire, MK43 0AL, UK; 2 Centre for Research in Biomedicine, Faculty of Health and Life Sciences, University of the West of England, Frenchay Campus, Coldharbour Lane, Bristol, BS16 1QY, UK; 3 Air Quality Management Resource Centre, University of the West of England, Bristol, Faculty of Enivronment and Technology, Coldharbour Lane, Bristol, BS16 1QY, UK; 4 Mouchel, No.1 Waterhouse Square, 138-142 Holborn, London, EC1N 2ST, UK

## Abstract

This paper describes an exploratory study of endotoxin emissions and dispersal from a commercial composting facility. Replicated samples of air were taken by filtration at different locations around the facility on 10 occasions. Measurements were made of endotoxin and associated culturable microorganisms. The inflammatory response of cell cultures exposed to extracts from the filters was measured. Endotoxin was detected in elevated concentrations close to composting activities. A secondary peak, of lesser magnitude than the peak at source was detected at 100-150 m downwind of the site boundary. Unexpectedly high concentrations of endotoxin were measured at the most distant downwind sampling point. Extracted endotoxin was found to stimulate human monocytes and a human lung epithelial cell line to produce significant amounts of pro-inflammatory cytokines. On a weight basis, endotoxin extracted from the composting source has a greater inflammatory cytokine inducing effect than commercial *E. coli *endotoxin.

## Background

The commercial composting industry in the United Kingdom (UK) has grown rapidly in response to the requirements of the Landfill Directive (EC/31/99). 0.06 million tonnes were composted in 1994 rising to nearly 2 million tonnes in 2004 [1]. 82% of the waste composted in 2003/04 was processed using open windrow technology which has limited control over emissions to the air [1]. The emission of culturable microorganisms, and their constituent parts, into the air from composting has been well documented [2-4], with agitation activities such as shredding, turning and screening shown to enhance the release of bioaerosols. The potential for public health to be adversely affected by bioaerosol emissions from composting has been identified [3,5]. In view of this, regulators in the UK require risk assessments to be completed for any licensed composting facility that has a sensitive receptor within 250 m of the facility boundary [6]. Whilst some progress has been made in the quantification of emissions and downwind dispersal of culturable microorganisms from composting facilities [7] relatively little is known about the concentrations in outdoor air of cell constituents such as endotoxin generated by composting.

Endotoxin is the biologically active lipopolysaccharide (LPS) that is a major constituent of the outer membrane of Gram-negative bacteria (GNB). Endotoxin is a potent proinflammatory agent that produces airway inflammation when inhaled. Elevated levels of endotoxin have been measured in various environments including cotton mills, grain storage and processing buildings, farm animal barns, sewage treatment plants and humidified work areas [8-12]. Inhalation of endotoxin at elevated concentrations have been associated with acute airway obstruction, hypersensitivity pneumonitis, chronic bronchitis and decreased lung function [13]. There have been several studies of occupational exposures and the health of workers in composting facilities. These have shown significant increases in airway and skin diseases than in controls. A study of Dutch compost workers [5] reported excess acute and sub-chronic non-immune inflammation in the upper airways which they suggested was induced by exposure to endotoxin and glucans. However, there have been very few studies of health in the general public around composting sites. Studies based on self-reporting criteria either found no association between respiratory symptoms and living near a composting site, or a significantly increased risk for a number of health complaints based on distance between home and the composting site [14].

This paper describes an exploratory study of endotoxin emissions and dispersal from a commercial composting facility. Replicated samples of air were taken by filtration at different locations around the facility on 10 occasions during 2007 and 2008. Measurements were made of endotoxin and associated culturable microorganisms. The inflammatory response of cell cultures exposed to extracts from the filters containing endotoxin was used as a preliminary step towards assessing the health significance of endotoxin release to the environment.

## Methods

Sampling took place at a large-scale green waste composting facility in central England. The site receives 25,000 tonnes green waste per annum, sourced from kerbside collection and civic amenity sites. Shredding and screening is carried out using dedicated machinery, whilst windrow turning is carried out using a front-loader. The sampling procedures have been described previously [15]. Briefly, samples were taken using SKC sampling pumps operated at a flow rate of 2.0 ± 0.1 L min^-1 ^and attached to SKC IOM particulate sampling heads, loaded with IOM multi-dust plastic cassettes and positioned at a height of 1.7 m. Each sample cassette contained a sterile 0.8 μm pore size polycarbonate filter. Six pumps were run simultaneously at each location for 30 min to capture microbial cells (n = 3) and endotoxin (n = 3). Samples were taken upwind of the facility, within the operational area, at the site boundary and at locations downwind of the boundary (50, 80, 100, 150, 180 and 280 m). Samples were taken at times of site activity (shredding, screening and turning of waste) or no activity (e.g. lunchtime). The site was sampled 10 times from Autumn 2007 to Summer 2008 at approximately monthly intervals. Methods for microbiological enumeration have been described previously [7,15]. Briefly, filtered cells were suspended in buffer solution, diluted and plated onto selective media for *Aspergillus fumigatus*, actinomycetes and Gram-negative bacteria. For endotoxin measurement, filters were extracted according to the methods described by Spaan et al. [16]. The extraction solution was then transferred into pyrogen-free tubes and centrifuged at 1000× g for 15 minutes. Supernatant was collected, vortexed and aliquots examined using the PyroGene rFC Endotoxin detection kit (Lonza Wokingham Limited, UK). To assess the potential for the environmental endotoxin to induce inflammatory responses, extracted endotoxin solutions were added to cultures of human airway epithelial and monocyte cells. After 24 h of incubation, the cell culture supernatants were harvested and stored at -80°C until analysis for the release of interleukin (IL)-6 and IL-8 into the supernatants by the DuoSet^® ^sandwich enzyme-linked immunosorbent assay (ELISA) kits according to the manufacturer's instructions (R&D Systems, Abingdon, UK).

## Results and discussion

**Figure 1:**
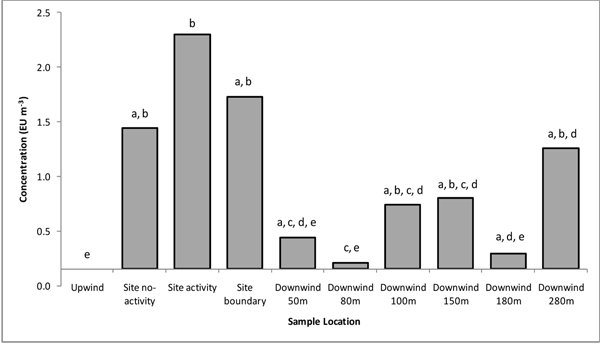
**Endotoxin concentrations presented in EU m^-3^**. Bar represents geometric mean, annotations present groups of statistical significance (p =< 0.05). E.g. Within group 'a' there is no statistical difference, while groups 'a' and 'b' are significantly different from each other.X-axis crosses at lower limit of detection, 0.152 EU m^-3^.

**Figure 2:**
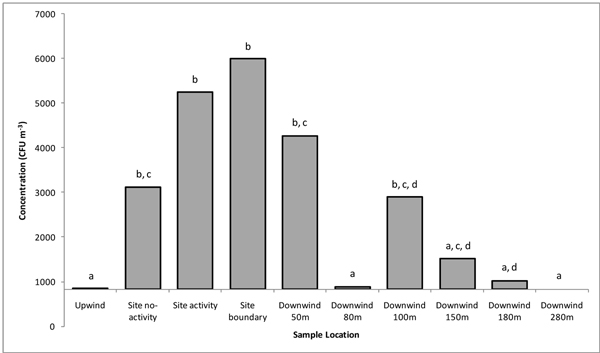
***Aspergillus fumigatus *concentrations presented in CFU m^-3^**. Bar represents geometric mean, annotations present groups of statistical significance (p =< 0.05). E.g. Within group 'a' there is no statistical difference, while groups 'a' and 'b' are significantly different from each other. X-axis crosses at lower limit of detection, 833 CFU m^-3^.

Endotoxin was not detected in samples taken upwind of the facility but was consistently detected in on site samples, both during periods of composting processing and periods where there was no site activity. Measurements downwind of the site showed a pattern of peaks and troughs (secondary peaks at 100-150 m and 280 m)(Figure 1). Culturable bioaerosol concentrations were typically below the limit of detection in upwind samples except for actinomycetes which are a significant component of the background microbiota of ambient air at this location. The highest concentrations were measured close to on-site composting activities and as with endotoxin, on-site concentrations of bioaerosols were consistently lower during periods when site activity ceased (example for *A. fumigatus *in Figure 2). Culturable bioaerosol concentrations decreased to 80 m followed by a second peak at 100 m. In contrast to the endotoxin findings there was no secondary peak for culturable bioaerosols at 280 m.

There were similarities in the patterns of emission and dispersal of culturable bioaerosols and endotoxin (Figures 1 and 2). Bioaerosol emission peaks associated with composting operations have previously been described by Taha *et al *[7] and Albrecht *et al *[1] for culturable microorganisms. On-site concentrations of all bioaerosols were consistently lower during periods when site activity ceased, confirming that agitation activities have a significant effect on emission. In all cases bioaerosol concentrations decreased sharply with downwind distance from the emissions source to the sampling point 80 m downwind of the site boundary. The rapid dispersion of bioaerosols downwind of composting facilities has been reported in previous studies [17]. All data sets showed a second peak in bioaerosol concentrations at 100-150 m. This phenomenon was not expected. It is possible this is a local effect caused by site topography/features which may influence plume dynamics (there is a bund and tree line close to this sampling location). However, further sampling at another composting site has produced similar findings (data not shown). The detection of endotoxin at elevated quantities up to 280 m from the site boundary was unexpected and to the authors' knowledge, has not been reported previously. This could be caused by a second source of endotoxin or a transport mechanism that is different to that of cellular bioaerosols. Quantities of endotoxin measured on site were consistently below 50 EU m^-3^, a suggested threshold level for occupational exposure in the Netherlands [18]. Extracted endotoxin induced inflammatory cytokine release from human cell cultures in a dose-dependent manner. However, cytokine release was minimal at concentrations less than ~50 EU m^-3^, supporting this level as a cut-off value for potential health effects. To our knowledge, there are no other reports of extracted endotoxin from such facilities being investigated for biological effects [19].

## Conclusion

1. The release and dispersal of endotoxin from composting operations has been quantified for the first time. Endotoxin was not detected in upwind samples but was detected consistently in on site samples confirming composting as a source. Concentrations were significantly higher during composting activities compared to periods of no activity. Downwind measurements showed a pattern of peaks and troughs suggesting complex dispersal dynamics. Detection of elevated concentrations at distance from the site boundary was unexpected but it was not possible to confirm that composting was its source.

2. Extracted endotoxin (>50 EU/m^3^) can induce significant inflammatory cytokine production from human cells. Endotoxin levels below 50 EU/m^3 ^did not induce a significant inflammatory response and this value might be used as a tentative limit for airborne endotoxin at these sites.

## Note

The peer review of this article can be found in Additional file 1.

## Competing interests

The authors declare that they have no competing interests.

## Authors' contributions

Lewis Deacon - in overall charge of the sampling programme and microbiological analysis, significant authorship inputs; Louise Pankhurst - significant contributor to the sampling design, principal microbiological analyst, statistical inputs and significant authorship inputs; Jian Liu, - endotoxin analysis and data analysis; Gillian Drew - significant contributor to sampling design on composting facilities; Enda Hayes - significant contributor to sampling design and advisor on wind tunnel sampling; Simon Jackson - principal investigator for the endotoxin component; James Longhurst - significant contributor to sampling design; Philip Longhurst, - contributions to sampling design and critical review of manuscript; Simon Pollard - substantial contribution to the conception of the study and final approval of the submitted version; Sean Tyrrel - principal investigator and significant authorship role.

## Supplementary Material

Additional file 1 Peer review Click here for file

## References

[B1] Slater R Davies P Gilbert EJ The State of Composting in the UK 2003/04 2005 The Composting Association. UK

[B2] Albrecht A Fischer G Brunnemann-Stubbe G Jäckel U Kämpfer P Recommendations for study design and sampling strategies for airborne microorganisms, MVOC and odours in the surrounding of composting facilities Int J Hyg Environ Health 2008 211 1-2 121 131 17765659 10.1016/j.ijheh.2007.05.004

[B3] Swan JRM Kelsey A Crook B Occupational and Environmental Exposure to Bioaerosols from Composts and Potential Health Effects - A Critical Review of Published Data 2003 Health and Safety Executive. UK

[B4] Wheeler PA Stewart I Dumitrean P Donovan B Health Effects of Composting. A study of three compost sites and review of past data 1999 The Environment Agency. UK

[B5] Douwes J Thorne P Pearce N Heederik D Bioaerosol health effects and exposure assessment: progress and prospects Annals of Occupational Hygiene 2003 47 3 187 200 12639832 10.1093/annhyg/meg032

[B6] Environment Agency Monitoring the Environmental Impact of Waste Composting Plants. R&D Technical Summary P1-216 2001 Environment Agency. Bristol, UK

[B7] Taha MPM Drew GH Tamer A Hewings G Jordinson GM Longhurst PJ Pollard SJT Improving bioaerosol exposure assessments of composting facilities - Comparative modelling of emissions from different compost ages and processing activities Atmospheric Environment 2007 41 4504 4519

[B8] Donham K Haglind P Peterson Y Rylander R Belin L Environmental and health studies of farm workers in Swedish swine confinement buildings British journal of industrial medicine 1989 46 1 31 37 2920141 10.1136/oem.46.1.31 PMC1009719

[B9] Palchak RB Cohen R Ainslie M Hoerner CL Airborne endotoxin associated with industrial-scale production of protein products in gram-negative bacteria American Industrial Hygiene Association journal 1988 49 420 421 3271389 10.1080/15298668891379990

[B10] Laitinen S Nevalainen A Kotimaa M Liesivuori J Martikainen PJ Relationship between bacterial counts and endotoxin concentrations in the air of wastewater treatment plants Applied and environmental microbiology 1992 58 11 3774 3776 10.1128/aem.58.11.3774-3776.1992 PMC183174 1482197

[B11] Rylander R Donham KJ Hjort C Brouwer R Heederik D Effects of exposure to dust in swine confinement buildings--a working group report Scandinavian journal of work, environment & health 1989 15 5 309 312 2799315 10.5271/sjweh.1846

[B12] Kennedy SM Christiani DC Eisen EA Wegman DH Greaves IA Olenchock SA Ye TT Lu PL Cotton dust and endotoxin exposure-response relationships in cotton textile workers The American review of respiratory disease 1987 135 1 194 200 3800146 10.1164/arrd.1987.135.1.194

[B13] Flaherty DK Deck FH Cooper J Bishop K Winzenburger PA Smith LR Bynum L Witmer WB Bacterial endotoxin isolated from a water spray air humidification system as a putative agent of occupation-related lung disease Infection and immunity 1984 43 1 206 212 10.1128/iai.43.1.206-212.1984 PMC263411 6690401

[B14] Herr CEW zur Nieden A Jankofsky M Stilianakis NI Effects of bioaerosol polluted air on airways of residents: A cross sectional study Occupational and Environmental Medicine 2003 60 5 336 10.1136/oem.60.5.336 PMC1740528 12709518

[B15] Taha MPM Drew GH Longhurst PJ Smith R Pollard SJT Bioaerosol releases from compost facilities: Evaluating passive and active source terms at a green waste facility for improved risk assessments Atmospheric Environment 2006 40 1159 1169

[B16] Spaan S Heederik DJ Thorne PS Wouters IM Optimisation of airborne endotoxin exposure assessment: effects of filter type, transport conditions, extraction solutions, and storage of samples and extracts Applied Environmental Microbiology 2007 73 19 6134 5143 10.1128/AEM.00851-07 PMC2075030 17675430

[B17] Swan JRM Crook B Gilbert EJ Microbial emissions from composting sites Issues in Environmental Science and Technology 2002 18 73 101

[B18] Liebers V Brüning T Raulf-Heimsoth M Occupational endotoxin exposure and possible health effects on humans American Journal of Industrial Medicine 2006 49 474 491 16586405 10.1002/ajim.20310

[B19] Liebers V Raulf-Heimsoth M Brüning T Health effects due to endotoxin inhalation (review) Archives of Toxicology 2008 82 203 210 18322674 10.1007/s00204-008-0290-1

