# Supplementary material for: Endotoxin emissions from commercial composting activities
Source: Environ Health. 2009 Dec 21;8(Suppl 1):S9. doi: 10.1186/1476-069X-8-S1-S9 (PMC2796505; doi:10.1186/1476-069X-8-S1-S9)
Supplement: Additional file 1 — Peer review [file 1476-069X-8-S1-S9-S1.docx]

| Article ref no. | A1471-2105X SZ S9 |
| --- | --- |
| Title | Endotoxin emissions from commercial composting activities |
| Author(s) | Deacon, L.J., Pankhurst, L.J., Liu, J., Drew, G.H., Hayes, E.T., Jackson, S.K., Longhurst, J.W.S., Longhurst, P. J., Pollard, S.J.T, and Tyrrel, S.F. |

**Ref#1**

**General comments:**

The paper reports half-hour endotoxin measurements taken in a roughly monthly time series around a commercial composting plant. Endotoxin extracts were then used to dose cells ex vivo and measure cytokine release. The paper combines a relatively simple endotoxin sampling campaign and toxicity study which provides preliminary evidence of endotoxin emissions from a commercial composting. This data is novel in the UK and provides some tentative conclusions on endotoxin emissions and toxicity.

**Major compulsory revisions:**

None

**Minor essential revisions:**

There are some limitations to the study and the paper. Small additions to the paper may resolve many of the current issues. For example, clarifying details of the sampling campaign will provide the reader with context for these interesting results and tentative conclusions (sampling times, maps, surrounding area and buildings, weather conditions, etc). Similarly, more detail on the methods for the toxicological study is required.

Specific Recommendations for Changes:

Reference 12 is very outdated – an updated reference on PM is recommended such as (or similar):

Russell, A. G. & Brunekreef, B. A Focus on Particulate Matter and Health. Environmental Science & Technology 43, 4620 (2009).

Please give sampling details to report time of day, map of sampling location, weather/season data, wind speed and direction on sampling days).

Please state whether the six parallel samples were taken at the same location or at all locations at the same time.

Please report details of the ELISA kit.

Please elaborate on the conclusions.

**Discretionary revisions:**

None

**Ref#2**

**General comments:**

Very good short paper recommended for publication with minor revision.

**Minor essential revisions:**

Provide details of ELISA kit/method

**Response to referees’ comments**

**Ref#1**

**Minor essential revisions:**

Specific Recommendations for Changes:

1. Reference 12 is very outdated – an updated reference on PM is recommended such as (or similar):

Russell, A. G. & Brunekreef, B. A Focus on Particulate Matter and Health. Environmental Science & Technology 43, 4620 (2009).

Response to comment 1. The suggested reference is a very recent and authoritative editorial on the significance of PM exposure to human health. It makes no reference to endotoxin however. We therefore assume that referee#1 was not comfortable with the sentence relating to reference 12 which reported on a study in which a health impact was correlated with exposure to endotoxin but not to dust. We did not intend to give the impression that we are diminishing the health significance of PM. We have therefore removed this ambiguous sentence.

2. Please give sampling details to report time of day, map of sampling location, weather/season data, wind speed and direction on sampling days).

Response to comment 2. We agree that this information is relevant to the paper. However the instructions for these short papers specified 2 figures/tables. As we have used our allocation of 2 figures for the presentation of results, there is no room for the addition of a site map and a table detailing the sampling conditions. We are happy to add these if the editor requests this.

3. Please state whether the six parallel samples were taken at the same location or at all locations at the same time.

Response to comment 3. We have clarified this in the text.

4. Please report details of the ELISA kit.

Response to comment 4. Details of the ELISA kit have been added to the methodology.

5. Please elaborate on the conclusions.

Response to comment 5. Additional detail has been added to the conclusions

**Ref#2**

**Minor essential revisions:**

1. Provide details of ELISA kit/method

Response to comment 1. Details now included in the methodology.

**Additional modification to the paper**

Preliminary statistical analysis had been carried out on the survey dataset at the time of writing the paper. Since then more detailed analysis has been completed. Figures 1 and 2 have been modified to reflect the new analysis. These modifications have not affected the conclusions of the paper.
